# Constitutive PSGL-1 Correlates with CD30 and TCR Pathways and Represents a Potential Target for Immunotherapy in Anaplastic Large T-Cell Lymphoma

**DOI:** 10.3390/cancers13122958

**Published:** 2021-06-12

**Authors:** Beatrice Belmonte, Valeria Cancila, Alessandro Gulino, Mohsen Navari, Walter Arancio, Paolo Macor, Andrea Balduit, Sara Capolla, Gaia Morello, Davide Vacca, Ines Ferrara, Giorgio Bertolazzi, Carmela Rita Balistreri, Paolo Amico, Federica Ferrante, Antonino Maiorana, Tiziana Salviato, Pier Paolo Piccaluga, Alessandro Mangogna

**Affiliations:** 1 Tumor Immunology Unit, Department of Health Sciences, University of Palermo, 90134 Palermo, Italy; beatrice.belmonte@unipa.it (B.B.); valeria.cancila@unipa.it (V.C.); alessandro.gulino@cogentech.it (A.G.); gaia.morello@unipa.it (G.M.); davide.vacca@unipa.it (D.V.); ines.ferrara@unipa.it (I.F.); giorgio.bertolazzi@unipa.it (G.B.); federica.ferrante@unipa.it (F.F.); 2 Department of Medical Biotechnology, School of Paramedical Sciences, Torbat Heydariyeh University of Medical Sciences, Torbat Heydariyeh 95196 33787, Iran; navarim2@thums.ac.ir; 3 Research Center of Advanced Technologies in Medicine, Torbat Heydariyeh University of Medical Sciences, Torbat Heydariyeh 95196 33787, Iran; 4 Bioinformatics Research Group, Mashhad University of Medical Sciences, Mashhad 91766 99199, Iran; 5 Advanced Data Analysis Group, Fondazione Ri.MED, 90133 Palermo, Italy; warancio@fondazionerimed.com; 6 Department of Life Sciences, University of Trieste, 34127 Trieste, Italy; pmacor@units.it (P.M.); abalduit@units.it (A.B.); scapolla@units.it (S.C.); 7 Department of BioMedicine, Neuroscience, and Advanced Diagnostics (Bi.N.D.), University of Palermo, 90134 Palermo, Italy; carmelarita.balistreri@unipa.it; 8 Department of Pathology, Cannizzaro Hospital, 95126 Catania, Italy; paolo.amico@aoec.it; 9 Department of Medical and Surgical Sciences for Children and Adults, University Hospital of Modena and Reggio Emilia, 41121 Modena, Italy; antonino.maiorana@unimore.it (A.M.); salviato.tiziana@aou.mo.it (T.S.); 10 Department of Experimental, Diagnostic, and Specialty Medicine, University of Bologna, 40126 Bologna, Italy; pierpaolo.piccaluga@unibo.it; 11 Section of Genomics and Personalized Medicine, Istituto Euro-Mediterraneo di Scienza e Tecnologia (IEMEST), 90139 Palermo, Italy; 12 Department of Pathology, School of Medicine, Jomo Kenyatta University of Agriculture and Technology, 00622 Juja, Kenya; 13 Institute for Maternal and Child Health, IRCCS (Istituto di Ricovero e Cura a Carattere Scientifico) “Burlo Garofolo”, 34137 Trieste, Italy

**Keywords:** PSGL-1, PTCL, ALCL, ALK, CD30, TCR, immunotherapy

## Abstract

**Simple Summary:**

P-selectin glycoprotein ligand-1 (PSGL-1), coded by the *SELPLG* gene, is the major ligand of selectins and plays a pivotal role in tethering, rolling and extravasation of immune cells. PSGL-1 involvement in core molecular programs, such as SYK, PLCγ2, PI3Kγ or MAPK pathways, suggests additional functions beyond the modulation of cell trafficking. Recently, several studies identified a novel mechanism responsible for PSGL-1-mediated immune suppression in the tumor microenvironment and proved a novel concept of PSGL-1 as a critical checkpoint molecule for tumor immunotherapy. The immunotherapeutic approach has gained an ever-growing interest in the treatment of several hematological malignancies, and in particular, novel targets for immunotherapy are still highly sought-after in T-cell lymphomas. Based on our results obtained through gene expression profiling and immunohistochemical analysis, PSGL-1, already suggested as a potential target in multiple myeloma humoral immunotherapy, could be considered noteworthy among the candidates.

**Abstract:**

Due to the high expression of P-selectin glycoprotein ligand-1 (PSGL-1) in lymphoproliferative disorders and in multiple myeloma, it has been considered as a potential target for humoral immunotherapy, as well as an immune checkpoint inhibitor in T-cells. By investigating the expression of *SELPLG* in 678 T- and B-cell samples by gene expression profiling (GEP), further supported by tissue microarray and immunohistochemical analysis, we identified anaplastic large T-cell lymphoma (ALCL) as constitutively expressing SELPLG at high levels. Moreover, GEP analysis in CD30+ ALCLs highlighted a positive correlation of *SELPLG* with *TNFRSF8* (CD30-coding gene) and T-cell receptor (TCR)-signaling genes (*LCK, LAT, SYK* and *JUN*), suggesting that the common dysregulation of TCR expression in ALCLs may be bypassed by the involvement of PSGL-1 in T-cell activation and survival. Finally, we evaluated the effects elicited by in vitro treatment with two anti-PSGL-1 antibodies (KPL-1 and TB5) on the activation of the complement system and induction of apoptosis in human ALCL cell lines. In conclusion, our data demonstrated that PSGL-1 is specifically enriched in ALCLs, altering cell motility and viability due to its involvement in CD30 and TCR signaling, and it might be considered as a promising candidate for novel immunotherapeutic approaches in ALCLs.

## 1. Introduction

P-selectin glycoprotein ligand-1 (PSGL-1) represents the major ligand for selectins (Platelets, Leukocytes and Endothelium selectins, or P-, L- and E-selectins), playing a relevant role in regulating the tethering, rolling and extravasation of cellular components of the immune system from peripheral blood to inflamed tissues [1,2,3]. The molecule is broadly distributed on polymorphonuclear leukocytes, monocytes, activated platelets and T-lymphocytes, as well as plasma cells [4].

PSGL-1, encoded by the *SELPLG* gene, is a homodimeric disulfide-linked glycoprotein with post-translational modifications responsible for the different affinity to P-, L- and E-selectins [5,6]. During the rolling process, several PSGL-1 proteins cluster in lipid rafts, and their cytoplasmic domains transduce signals via the spleen tyrosine kinase (SYK), leading to secretion of cytokines and activation of membrane integrins, which, in turn, promote extravasation [7].

PSGL-1 has also been indicated as a signaling molecule involved in core molecular programs such as SYK, PLCγ2, PI3Kγ or MAPK signal transduction pathways, suggesting other roles of PSGL-1 besides immune cell trafficking [7,8,9]. PSGL-1 has been shown to induce caspase-independent apoptosis in activated T-cells [10], suppress the late-phase immune response in lymph nodes via regulatory T-cells [11] and stimulate a tolerogenic function of dendritic cells, resulting in the regulation of the immune response [12]. Moreover, it has emerged as an immune checkpoint regulator promoting T-cell exhaustion [13] reasonably through the up-regulation of programmed cell death protein-1 (PD-1) [14] and IL-2 down-regulation [15]. Emerging evidence has unveiled an interaction between V-domain immunoglobulin suppressor of T-cell activation (VISTA), a newly proposed PD-1 homolog [16], and PSGL-1 in the suppression of immune responses selectively in acidic microenvironment as that found in tumors [17,18]. Besides its commonly dissected functions, a novel role of PSGL-1 has recently emerged as a restriction factor for virus infection, inhibiting HIV-1 [19,20] and SARS-CoV-2 [21] particle attachment to target cells. Due to its previously unknown selectin-independent roles and its ability to induce T-cell exhaustion, PSGL-1 has also been proposed as a potential target for immune modulation [22] and humoral immunotherapy [23] because it is highly expressed in lymphoproliferative disorders with plasmocytic differentiation and in multiple myelomas (MMs) as well [23,24,25].

The present investigation aims at evaluating the expression of SELPLG and its potential molecular interactions in T-cell lymphomas. In particular, we focused our interest on peripheral T-cell lymphomas (PTCLs), a heterogeneous group of aggressive non-Hodgkin lymphomas (NHLs), distinguishing among the most common subtypes of PTCLs: PTCLs, not otherwise specified (PTCLs, NOS), anaplastic large-cell lymphomas (ALCLs) and angioimmunoblastic T-cell lymphomas (AITLs) [26,27]. No effective targeted therapies have been yet identified for PTCLs, except for Brentuximab vedotin, an antibody-drug conjugated for targeting the TNF-receptor CD30 [28], and Rituximab, currently used for the treatment of CD20-positive PTCLs [29,30].

For these reasons, we analyzed the expression and the molecular correlations of PSGL-1 in PTCL samples, investigating its potential suitability as a functional target.

## 2. Materials and Methods

### 2.1. Sample Selection

This study was carried out according to the clinical standards of the 1975 and 1983 Helsinki Declaration and approved by the University Hospital of Palermo Ethical Review Board (approval number 09/2018).

In total, 110 ALCL cases and 50 PTCL, NOS cases were collected from either the Institute of Hematology and Medical Oncology “L. and A. Seràgnoli” at the University of Bologna, or the Human Pathology Section of A.O.U.P. “Paolo Giaccone” at the University of Palermo. All formalin-fixed paraffin-embedded tissue samples deriving from lymph nodes had been previously processed for routine histopathological diagnosis. Hematoxylin–eosin (H&E) stained sections that were 4-µm thick from all tissue blocks were analyzed by at least 2 experienced hematopathologists.

### 2.2. Analysis on Tissue Microarrays

For tissue microarrays (TMAs), 2 mm tissue cores were transferred and deposited into pre-manufactured paraffin recipient blocks (Tissue-Tek^®^, Sakura, The Netherlands). After transferring all cores, the fusion of the recipient block was achieved by heating the blocks at 60 °C for 1h. Subsequently, 5-µm thick tissue sections were cut from the TMAs, and the first section was H&E stained to verify the tissue patterns and to identify lost cores in the section.

### 2.3. Immunohistochemical Analysis

Immunohistochemistry (IHC) was performed using a polymer detection method. Briefly, tissue samples were fixed in 10% buffered formalin and paraffin-embedded. The, the 4-µm thick tissue sections were deparaffinized and rehydrated.

The antigen unmasking technique was performed using Novocastra Epitope Retrieval Solutions pH = 9 or pH = 6 in a PT Link Dako pre-treatment module at 98 °C for 30 min. The sections were then brought to room temperature (RT) and washed in PBS. After neutralization of the endogenous peroxidase with 3% H_2_O_2_ and Fc blocking by a specific protein block (Novocastra, Newcastle, UK), the samples were incubated for 1h with the primary antibodies at RT. Primary antibodies were listed in Table 1.

Staining was revealed by polymer detection kit (Novocastra) and 3-Amino-9-ethylcarbazole (AEC) Dako substrate chromogen. The slides were counterstained with Harris hematoxylin (Novocastra). Slides were analyzed under a Zeiss Axioscope A1, and microphotographs were collected using a Zeiss Axiocam 503 Color camera with the Zen 2.0 Software (Zeiss, Oberkochen, Germany).

IHC was evaluated based on the intensity of staining and scored as grade 0 (negative), grade 1 (weak), grade 2 (moderate) or grade 3 (strong).

### 2.4. Gene Expression Profiling Analysis

Gene expression profiling (GEP) was generated and analyzed as previously reported [31]. Briefly, the total RNA was extracted from cryopreserved biopsy samples of PTCLs or normal T-cells; fragmented biotinylated cRNA was then hybridized to HG-U133 2.0 plus microarrays (Affymetrix Inc., Santa Clara, CA, USA). Gene expression values were determined by MAS 5 algorithm in GCOS 1.2; Affymetrix, Inc.

A detailed list of samples used in GEP analysis has been reported in Table 2.

Raw gene expression data were extracted from the GEO Database (Gene Expression Omnibus of the National Center for Biotechnology Information-NCBI), previously generated at our or other Institutions. T-cell setting GSE14879, GSE19069, GSE6338, and B-cell setting GSE4732, GSE12453, GSE12195, GSE35426, GSE12195, GSE16455, GSE24080 were included.

### 2.5. Cell Lines and Antibodies

ALCL cell lines, including KARPAS-299, L-82, MAC, SU-DHL-1 and TS, were cultured in RPMI-1640 medium (Sigma-Aldrich, Darmstadt, Germany) supplemented with 10% fetal bovine serum (FBS; Sigma-Aldrich). The endothelial cell line EA.hy926 was cultured in Dulbecco’s Modified Eagle’s Medium (DMEM) supplemented with 2 mM L-glutamine and 10% FBS.

Primary antibodies used in cellular and functional assays were listed in Table 3.

### 2.6. Flow Cytometry

ALCL cell lines (5 × 10^5^) were incubated with 5 µg/mL of KPL-1 or TB5 antibodies for 1h at 37 °C followed by incubation with 30 µg/mL of FITC-labeled anti-mouse IgG. For carrying out these measurements, 10,000 events were acquired using the FACSCalibur (Becton Dickinson, Franklin Lakes, NJ, USA) flow cytometer, and the data were analyzed by CELLQuest software (Becton Dickinson).

### 2.7. Adhesion Assay

EA.hy926 endothelial cells (5 × 10^4^/well) were seeded onto a 96-well cell culture plate in DMEM supplemented with 2 mM L-glutamine and 10% FBS. The day after, SU-DHL-1 (1 × 10^5^ cells) were stained with the VybrantTM DiI cell-labeling solution (GE Healthcare, Chicago, IL, USA), incubated for 1h with KPL-1, TB5 or isotype control antibody (2.5 µg/mL) and then incubated with EA.hy926 endothelial cells for 1 h at 37 °C. Unbound cells were removed by 3 washing steps, and the fluorescence of bound cells was detected with an Infinite 200 (TECAN) multimode plate reader.

### 2.8. Cell Viability and Apoptosis

SU-DHL-1 cells (2 × 10^5^ cells) were incubated with KPL-1 or TB5 antibodies (2.5 µg/mL) for 48 h at 37 °C under shaking conditions. The amount of residual viable cells was determined using the MTT assay, and the percentage of dead cells was calculated as: ((test release − spontaneous release)/(total release − spontaneous release)) × 100.

Antibody-dependent cellular cytotoxicity (ADCC) was performed as previously described [23] with some modifications. In detail, human peripheral blood mononuclear cells (PBMCs) were separated from the blood of healthy donors by Ficol-Hypaque density gradients. Cells were washed once in PBS and then incubated with the fluorescent cytoplasmic dye 5-(and-6)-carboxyfluorescein diacetate, succinimidylester (Invitrogen, Waltham, MA, USA), using 25 µL of a 10 µM solution/10^6^ cells for 7 min in a 37 °C water bath. ALCL cells were harvested, washed and incubated with 2.5 µg/mL of either KPL1 or TB5 monoclonal antibody (mAb) Ig for 30 min at 4°C. Target cells were then mixed with human effector cells at an effector-to-target ratio of 40:1 and incubated for 6h in a humidified 37 °C, 5% CO_2_ incubator. The percentage of dead cells was evaluated by analyzing lactate dehydrogenase (LDH) release by Cytotox assay (Promega, Milan, Italy).

Complement-dependent cytotoxicity was assessed as previously described [32]. Briefly, 2 × 10^5^ cells were incubated with TB5 (2.5 µg/mL) in the presence or in the absence of anti-CD46, CD55 and CD59 (10 µg/mL) blocking antibodies to a final volume of 100 µL for 10 min at RT prior to the addition of normal human serum (25%) as a source of the complement system. After further incubation at 37 °C for 1 h, the number of residual viable cells was estimated using MTT assay, and the percentage of dead cells was calculated.

### 2.9. Statistical Analysis

All statistical analyses were performed using R statistical software (v 4.0.2). The differences in *SELPLG* expression among the samples were analyzed by *t*-test. The *t*-test *p*-values have been adjusted for multiple comparisons using the Benjamini–Hochberg method. Adjusted *p*-values < 0.05 have been considered significant. The differences of SELPLG expression in IHC among PTCL subgroups were analyzed by chi-square test. When the conditions of the chi-square test could not be satisfied due to sample sizing, Fisher’s exact probability method was used. The expression values of the genes directly correlated with *SELPLG* were extracted from GEP data. Correlation analysis of *SELPLG* with key genes was performed using Pearson’s correlation coefficient. *T*-test was used for the analysis of functional (adhesion and killing) studies where results were expressed as a mean of at least 3 independent experiments.

## 3. Results

### 3.1. SELPLG Gene and Its Coded Protein PSGL-1 Are Constitutively Expressed in ALCLs

We evaluated the expression of *SELPLG* in 678 samples, comprising malignant and non-malignant T- and B-cells, by GEP analysis.

**Figure 1:**
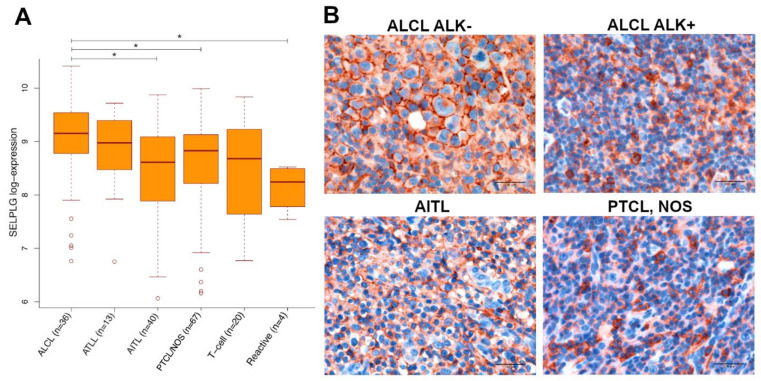
*SELPLG* differential expression in T-cell specimens and PSGL-1 expression in IHC analysis. (**A**) Histogram representing *SELPLG* gene expression in T-cell specimens using GEP data analyzed by Fisher’s protected least significant difference (PLSD) post hoc test. * = adjusted *p*-value < 0.05. (**B**) IHC analysis of PSGL-1 expression intensity in ALK- or ALK+ ALCL, AITL and PTCL, NOS specimens. Scale bar: 100 µm.

Evaluating T-cell setting, the expression of *SELPLG* resulted significantly higher in ALCLs (Figure 1A; Figure S1A,B) as compared with AITLs PTLCs, NOS, and reactive lymphoid tissues (adjusted *p*-value ≤ 0.05), whereas a borderline *p*-value (0.055) was obtained from the comparison between ALCLs and normal T-cells. No difference could be highlighted with ATLLs. Surprisingly, no statistically significant difference was determined in *SELPLG* gene expression between ALK+ and ALK- ALCL specimens (Figure S1C).

In the B-cell setting (Figure S2), the highest *SELPLG* gene expression was pointed out in plasma cells and related neoplasms. On the contrary, naïve B-cells and germinal center B-cells, along with their malignant counterparts, showed lower levels of *SELPLG* gene expression. Interestingly, classical Hodgkin lymphomas (CHLs) and T-cell/histiocyte-rich large B-cell lymphomas (THRLB-CLs) showed a high level of *SELPLG* expression.

We proceeded further by evaluating the in situ expression of SELPLG coded protein, PSGL-1, by IHC. According to this aim, we used a panel of TMAs encompassing 110 ALCLs and 50 PTCLs, NOS specimens (Table S1). By using a semiquantitative IHC approach that considered both intensity and distribution of the staining (Figure S3A), PSGL-1 resulted variably expressed in PTCLs, NOS samples, whilst an almost constant membrane expression has been detected in ALCL tissues (Figure S3B), showing a higher average expression intensity in comparison with AITLs and PTCLs, NOS (Figure 1B).

### 3.2. SELPLG Is Part of a Peculiar Transcriptional Network in ALCLs

**Figure 2:**
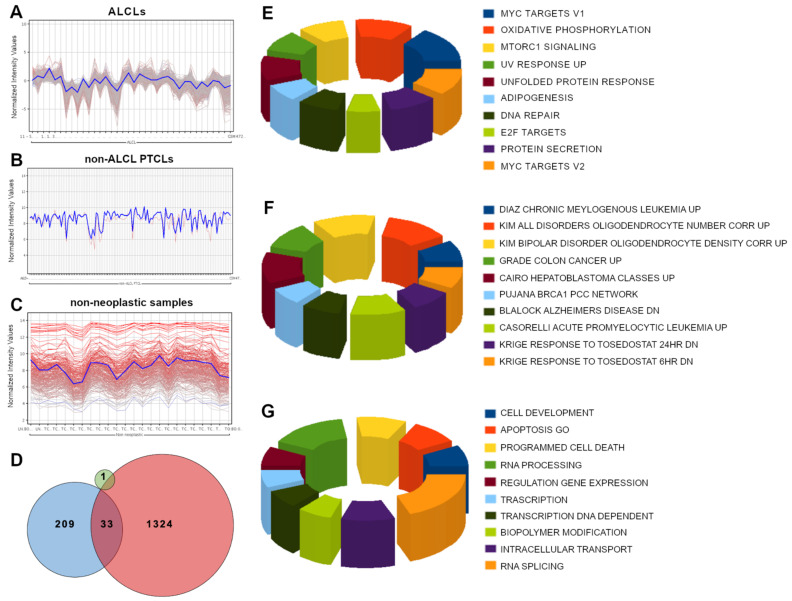
GEP analysis and GSEA results of the genes related to *SELPLG*. GEP analysis identified the presence of several genes showing a positive Pearson product-moment correlation coefficient (0.75–1) with *SELPLG* gene expression in ALCLs (**A**), non-ALCL PTCLs (**B**) and non-neoplastic samples (**C**). *SELPLG* gene presented 1358 significantly correlated genes in the ALCL setting (red), 242 in non-neoplastic samples (cyan) and 1 in non-ALCL PTCLs (green). In total, 1324 were exclusively correlated with the SELPLG gene within the ALCL setting (**D**). GSEA results of the genes related to *SELPLG* in ALCLs, comprising hallmark gene sets (**E**), C2 curated gene sets, chemical and genetic perturbations (**F**) and GO enrichment analysis (**G**).

GEP analysis was performed in order to inquire whether the *SELPLG* expression in ALCLs is linked to specific transcriptional networks, selecting the genes showing a positive Pearson product-moment correlation coefficient (from 0.75 to 1) with *SELPLG* expression in three different settings: ALCLs, PTCLs other than ALCLs, and non-neoplastic specimens (Figure 2A–C). Strikingly, *SELPLG* showed a high number of significantly correlated genes in ALCLs (1358 genes), whereas the number appeared considerably lower in non-neoplastic samples (242 genes) and non-ALCL PTCLs (1 gene); of these genes, 1324 were exclusively correlated with *SELPLG* within the ALCL setting, highlighting the existence of a peculiar transcriptional network comprising SELPLG (Figure 2D).

A parallel analysis was performed on the B-setting, pooling together CHLs and B-NHLs. As no significant correlates were retrieved at the previous stringency level, the analysis was repeated at a more permissive correlation coefficient of 0.50–1 (Figure S4A). 142 genes showed a correlation with *SELPLG* expression, but only 32 of these overlapped with the genes that emerged from the ALCL analysis and 6 with non-neoplastic samples (Figure S4B).

A detailed analysis of all the reported correlations can be found in Data S1.

### 3.3. Gene Set Enrichment Analysis and Gene Ontology Analysis Highlighted Several Cancer-Related Pathways within SELPLG Network in ALCLs

Gene Set Enrichment Analysis (GSEA) of the genes related to *SELPLG* in ALCL setting mainly detected genes involved in pathways and cellular functions specifically deregulated in cancer (Figure 2E,F), including MYC and E2F target gene sets, apoptosis and programmed cell death.

Gene Ontology (GO) analysis about the genes related to *SELPLG* (GO functional annotation clustering, standard conditions, in DAVID, https://david.ncifcrf.gov, accessed on 5 May 2021) (Data S2), reported the enrichment of very few GO annotations in the normal condition, mainly regarding innate immune response, defense response to virus and protein synthesis. By contrast, the GO annotations, including cell adhesion, proteasome-mediated catabolic processes, regulation of Wnt signaling, MAPK and many others, resulted more conspicuously enriched in ALCLs (Figure 2G).

### 3.4. SELPLG Expression Is Positively Correlated with TNFRSF8 Expression

**Figure 3:**
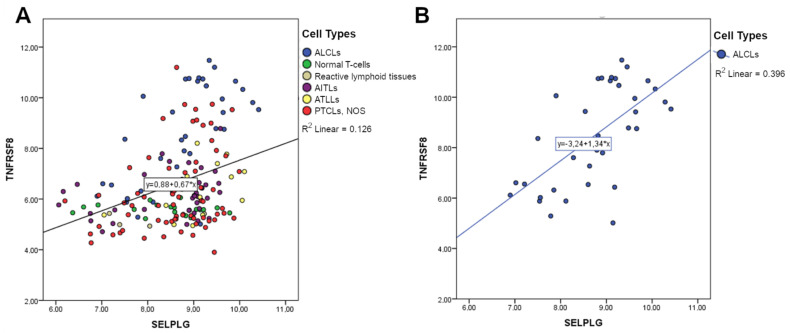
The correlation between *SELPLG* and *TNFRSF8* expression values. A comparison of *SELPLG* and *TNFRSF8* expression in all T-samples (**A**). A stronger positive correlation was highlighted when considering only ALCL samples (**B**). *p*-value < 0.01.

Since CD30 (also known as *TNFRSF8*) is an important marker of T/B-cell activation and a constant membrane antigen in ALCLs, we compared the expression of *SELPLG* and *TNFRSF8* in the whole T-cell setting, detecting a positive correlation by GEP analysis (Figure 3A). Interestingly, the positive correlation appeared to be stronger when considering only ALCL samples (Figure 3B). In order to understand whether the correlation could be exclusively T-cell specific, we extended the analysis to the B-cell setting, reporting a weaker positive correlation between the expressions of these two genes (Figure S5). These data strongly suggested that the expression of *SELPLG* and *TNFRSF8* are correlated.

In order to infer how *SELPLG* and *TNFRSF8* may be correlated, a network analysis was performed by GeneMANIA [33]. The study pointed out that six genes potentially correlated with both *SELPLG* and *TNFRSF8* (Figure S6), mainly through co-expression studies: *ITGAM* (integrin subunit alpha M, also called CD11b, regulating leukocyte adhesion and migration in inflammatory responses), *P2RY6* (pyrimidinergic receptor P2Y6, a G-protein coupled receptor, proposed as a mediator of inflammatory response), *TNFRSF4* (OX40 coding gene, an inhibitor of apoptosis involved in CD4+ T-cell response), *VCAN* (versican, a component of the extracellular matrix involved in cell adhesion, proliferation, migration, angiogenesis and tissue morphogenesis), *TNIP1* (whose gene products are regulators of NF-κB activation) and *TRAF1* (whose gene products mediate anti-apoptotic signals starting from TNF receptors). Overall, these genes are mainly involved in NF-κB, CD40 signaling and regulation of the immune response, according to GeneMANIA analysis (Data S3). 

### 3.5. SELPLG Signaling Correlates with TCR Signaling in ALCLs

**Figure 4:**
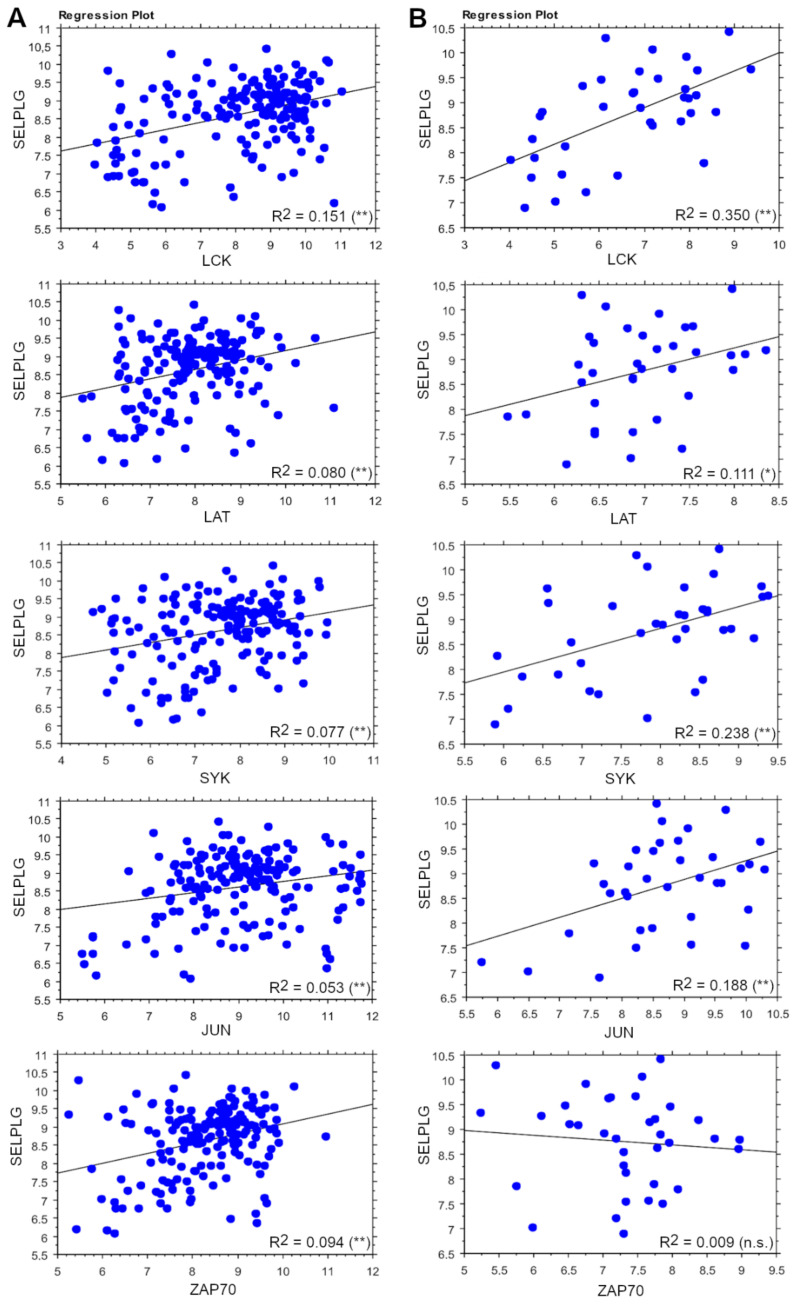
The correlation of the expression of *SELPLG* and TCR effector genes. The expression of *LCK, LAT, SYK, JUN* and *ZAP70* genes in correlation with that of *SELPLG* in the whole T-cell setting (**A**) and in ALCLs only (**B**). *p*-values: n.s. = not significant; * = < 0.05; ** = < 0.01.

Since an altered expression of several downstream effectors of TCR has been previously reported [34], the potential correlation of *SELPLG* with effector genes of the TCR program was evaluated in the whole T-cell setting and in the ALCL setting. Interestingly, in the T-cell setting, *LCK, LAT, SYK* and *JUN* showed a positive correlation with *SELPLG*, which appeared stronger when evaluating only ALCLs. On the contrary, *ZAP70* was correlated only in the whole T-cell setting but not in ALCLs (Figure 4). *FOS* showed a very weak correlation only in the T-cell setting, whilst *CARD11* and *BCL10* did not show any significant correlation; moreover, *NFATC1* resulted negatively related (Figure S7).

**Figure 5:**
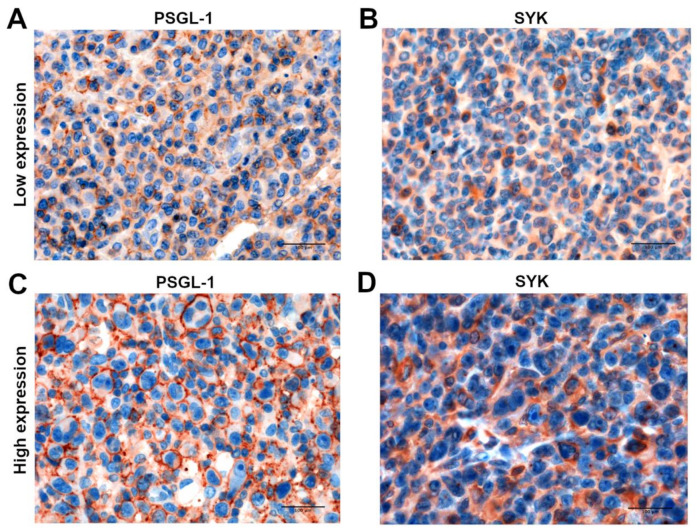
In situ IHC expression of PSGL-1 and SYK in prototypical ALCL cases. A similar pattern of expression was detected between PSGL-1 and SYK in ALCL cases by IHC assay: ALCL cases with a low expression level of PSGL-1 (**A**) showed also a lower expression of SYK (**B**), whilst highly expressing PSGL-1 samples (**C**) demonstrated comparable high levels of SYK (**D**). Scale bar: 100 µm.

A similar pattern of expression between SYK and PSGL-1 was also confirmed by IHC performed in ALCL prototypical cases. In fact, highly expressing PSGL-1 cases showed a higher expression of SYK and vice versa (Figure 5).

**Figure 6:**
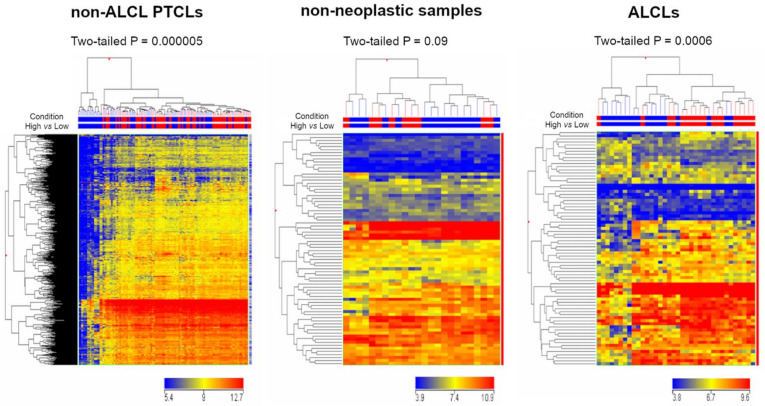
SELPLG gene expression correlates with TCR signaling. TCR: No significant enrichment at Gene Set Enrichment Analysis (GSEA), but correlation at clustering. Red: high expression; blue: low expression.

These data strongly suggested that SELPLG signaling correlates with TCR signaling in ALCLs (*p*-value ≤ 0.05) (Figure 6).

Finally, we investigated whether PSGL-1 may be activated in vivo in the context of ALCL, proving that its physiological ligands (P-, E- and L-selectins) were coherently present in prototypical cases showing a relatively low level of PSGL-1 (Figure S8). In particular, the diffuse L-selectin staining detected on the membranes of the neoplastic clones suggested a potential feedback loop of activation where the neoplastic clones showed diffusely positive staining for both PSGL-1 and L-selectin (Figure S8C).

### 3.6. In Vitro Analyses on ALCL Cell Lines Show That Anti-PSGL-1 Is Able to Induce Cell Cytotoxicity and Inhibit Cell Adhesion

Collected data evidenced the potential role of PSGL-1 as a tumor-associated marker of ALCL cell surface, representing a potential target for anti-PSGL-1 antibodies therapy.

**Figure 7:**
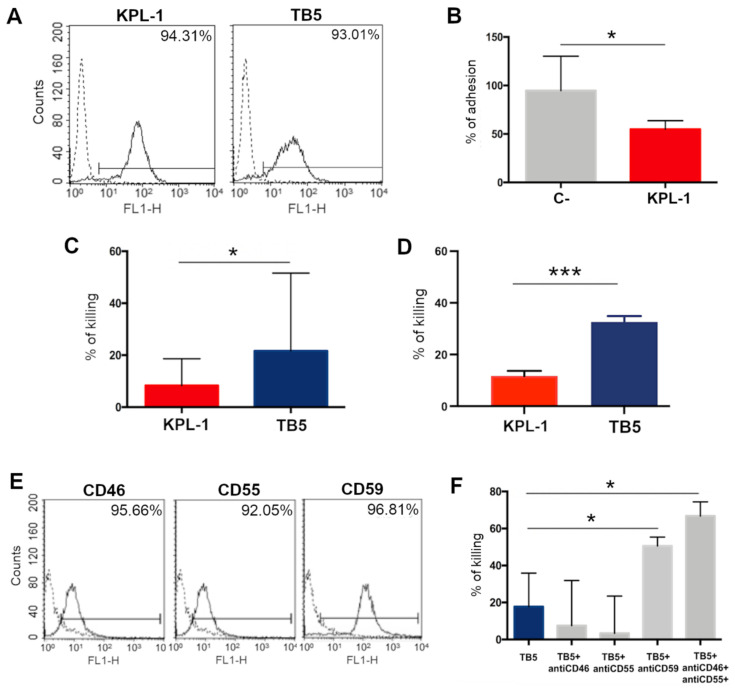
In vitro effect of anti-PSGL-1 antibodies on ALCL cell lines. (**A**) SU-DHL-1, a representative ALCL cell line, was analyzed by flow cytometry using two independent anti-PSGL-1 antibodies, KPL-1 and TB5, and a FITC-conjugated secondary antibody. (**B**) KPL-1 antibody was able to partially reduce SU-DHL-1 adhesion on EA.hy926 endothelial cell lines. (**C**) TB5 is able to induce direct cytotoxicity, analyzing residual cell viability after 48 h of incubation with SU-DHL-1 cells. (**D**) TB5, more than KPL-1, is able to induce ADCC of SU-DHL through PBMC activation and analyzing LDH release. (**E**) SU-DHL-1 cells evidenced the expression of CD46, CD55 and CD59 in FACS analysis after the incubation with specific antibodies and FITC-labeled secondary antibodies. (**F**) The binding of TB5 on the surface of ALCL cells is effective in inducing low levels of complement-dependent cytotoxicity using human serum as the source of complement. The percentage of lysed cells through CDC rises to significant values following blockage of the membrane complement regulatory proteins CD46, CD55 and CD59 expressed on SU-DHL-1 cells. *p*-values: * ≤ 0.05; *** ≤ 0.001.

Several ALCL cell lines (KARPAS-299, L-82, MAC-1, SU-DHL-1 and TS) were analyzed for their PSGL-1 surface expression by flow cytometry; two different anti-PSGL-1 antibodies (KPL-1 and TB5) detected a high protein expression in more than 80% of the cells (Figure S9). SU-DHL-1 was chosen as a representative prototype of ALCL cell lines (Figure 7A).

SU-DHL-1 adhesion on endothelial cell lines was tested, demonstrating that the anti-PSGL-1 neutralizing antibody KPL-1 is able to inhibit 45% of cell adhesion (Figure 7B). As opposed, TB5 was not able to affect the adhesion of cells.

The direct cytotoxicity of both antibodies in SU-DHL-1 was then evaluated by an MTT assay (Figure 7C), highlighting that, although variable, the direct cytotoxicity is mainly present using TB5, since around 20% of ALCL cells resulted killed by the treatment in an MTT test.

The binding of antibodies on ALCL cells could also induce the activation of the immune system, increasing the cytotoxicity effect induced by anti-PSGL1 molecules. For this reason, we tested the capacity of KPL-1 and TB5 to cause PBMC-induced ADCC of ALCL cells. TB5 resulted more efficiently than KPL-1, causing up to 33% and 12% of cell lysis, respectively (Figure 7D).

Finally, the complement-dependent cytotoxicity induced by anti-PSGL-1 antibodies was evaluated: KPL-1 was not able to induce complement activation on ALCL cell lines; on the contrary, TB5 activated the complement system and induced around 20% cell lysis, but, as expected, its activity was partially neutralized by the presence of membrane complement regulatory proteins on ALCL cells. Figure 7E shows the expression of CD46, CD55 and CD59, membrane complement regulatory proteins, on SU-DHL cells. In fact, in Figure 7F, the capacity of TB5 to induce complement activation of cancer cells is clearly evident, in particular when CD46/CD55/CD59 were neutralized by specific blocking antibodies not directly able to activate the complement system, causing up to 70% of cell lysis.

## 4. Discussion

ALCL is an aggressive non-Hodgkin type of PTCLs, accounting for about 3% of all lymphoid malignancies in adults and 10%–20% of pediatric lymphomas, as reported in the revised fourth edition of “Classification of Tumors of the Hematopoietic and Lymphoid Tissues” of the World Health Organization (WHO 2016/2017) [27]. ALCLs encompass different clinical entities that histologically share the presence of large pleomorphic T-cells expressing CD30 [27,35] and are usually classified according to the presence or absence of ALK chromosomal translocation [26,27], which confers a peculiar transcriptional profile to malignant cells [36].

At present, the first-line treatment for patients diagnosed with systemic ALCL is represented by CHOP (cyclophosphamide, doxorubicin, vincristine and prednisone) standard chemotherapy, but the recurrence of refractory or relapsed disease entailed the use of stem cell transplantation and the FDA approval of four novel single-agent treatments: pralatrexate, a folic acid antagonist, romidepsin and belinostat, two histone deacetylase inhibitors and Brentuximab vedotin, a drug-conjugated monoclonal antibody directed to CD30-expressing cells [37]. Over the last decades, the immunotherapeutic approach has gained an ever-growing interest in the treatment of several hematological malignancies [28,38] because the presence of circulating cancer cells and the expression of a rich repertoire of surface antigens make these kinds of diseases good candidates for monoclonal antibody (mAb)-based therapies. In particular, novel targets for immunotherapies are still highly sought-after in T-cell lymphomas. In our initial hypothesis, PSGL-1, which was already suggested as a potential target in MM humoral immunotherapy [23], could be noteworthy among the candidates.

To confirm our hypothesis, evaluating the presence of SELPLG in T and B-cell settings, we assessed that SELPLG is constantly more expressed in ALCL specimens both at the RNA level, according to GEP analyses, and at the protein level, as observed through the IHC in tissue microarray, in comparison with other T-cell lymphomas and controls. The absence of significant differences in *SELPLG* expression between ALK+ and ALK- ALCLs suggested its independence from ALK presence, leading us to believe that PSGL-1-based immunotherapy could be equally efficient in both subgroups, although usually ALK- ALCLs are the most difficult to treat [36]. Within the B setting, CHLs and THRLB-CLs interestingly showed higher expression since they share a reactive environment abundant in T-cells and activated lymphoid cells and the expression of CD30 [26,27].

The striking abundance of genes whose expression is related to *SELPLG* in ALCLs, differently from other tumoral or normal settings analyzed, seemed to be yet another confirmation of the role of this molecule in ALCLs, suggesting its involvement in a peculiar transcriptional network. In particular, both MYC and E2F target gene sets resulted as enriched and potential indicators of a sustained proliferative signal and clone survival in ALCLs [39,40], also supported by the enrichment of genes involved in transcription, mTORC1 signaling, unfolded protein response and protein secretion. Moreover, we observed increased oxidative phosphorylation, which may compensate for the increased energy requirements at the expense of genomic stability, justifying an increased presence of DNA repair genes and UV response gene expression. Unsurprisingly, GSEA also showed enrichment in genes implicated in apoptosis and programmed cell death, suggesting that antiproliferative and death mechanisms are still active in ALCLs, being potentially exploited during therapeutic approaches.

The GO analysis also reported that the enriched GO annotations for the genes related to *SELPLG* in ALCLs were more conspicuous than in normal T-cells, revealing a very rich plethora of functions mainly involving cell adhesion, energy production and metabolic processes, RNA metabolism, cell cycle regulation and signal transduction, among which we highlighted the TCR signaling pathway. In fact, an open debate in ALCLs regards the lack of a functional TCR, despite showing an activated phenotype, as confirmed by strong and uniform expression of CD30 [41,42]. Considering the enhanced expression of *SELPLG* in ALCLs and its fundamental functions in these lymphomatous cells, we hypothesized that the dysregulation of TCR might be bypassed (even if not exclusively) by the action of PSGL-1 in inducing proliferation and survival. Our assumption seemed to find confirmation when evaluating the correlation between *SELPLG* and TCR effector genes: surprisingly, *SELPLG* expression has been found to be positively correlated with *SYK, LCK, LAT, FOS* and *JUN.* Noteworthy, *ZAP70* is correlated only in normal T-cells, coherently with previous evidence reporting that SYK is able to substitute ZAP70 in transducing activator signaling [43]. These observations strongly suggest that PSGL-1 could be involved in the TCR activation of neoplastic clones mainly via the intracellular signaling of SYK, a central downstream effector of both TCR and PSGL-1 pathways.

Moreover, the action of PSGL-1 may be also triggered by the L-selectins expressed on the surface of the neoplastic clones, as we reported their abundance in IHC. Our data reported that P-selectin staining on the surface of intratumoral platelets is also common.

Another confirmation to our hypothesis was found in the strong relationship between *SELPLG* and *TNFRSF8*, reporting a correlation in all the settings analyzed, but primarily in ALCLs, suggesting once more its important role in these cells. As already mentioned, ALCL histotypes histologically share the presence of large pleomorphic T-cells that show a strong expression of CD30 as an activation marker usually expressed by T-cells and to a lesser extent by B-cells as well [35]. Interestingly, the presence of this correlation both in the T- and B-setting, even though the B-setting comprises a plethora of histotypes in which CD30 expression is not always reported, suggesting that their relation is not cell-intrinsic. According to GeneMANIA analysis, the network between SELPLG and CD30 coding genes appeared enriched in genes mainly involved in NF-κB, CD40 signaling and regulation of immune response, suggesting that the combined role of these genes might also influence the microenvironment.

Therefore, taking into account that CD30 can act as a positive regulator either of proliferation or apoptosis [42] depending on the microenvironment [44] and also that anti-CD30 therapies are currently in use [28], these data led us to hypothesize that immunotherapy using anti-PSGL-1 antibodies is worth exploring. To this purpose, the anti-PSGL-1 effect on ALCL cell lines was initially tested, demonstrating that antibodies against PSGL-1 were able to induce cell cytotoxicity or inhibit cell adhesion. In particular, apoptosis and complement-mediated cytotoxicity were induced by TB5. PSGL-1 needs to aggregate in discreet lipid rafts to promote its intracellular signaling [7,10,45] and its aggregation after antibody binding guarantees a high concentration of the Fc portion of antibodies on the cell surface, also triggering the activation of the classical pathway of the complement system and lysis of target cells. Based on this evidence, we can suppose that TB5 may be able to cause the capping of PSGL-1 on the surface of ALCL cell lines. On the contrary, as expected, the inhibition of cell adhesion was exerted only by the anti-PSGL-1 blocking antibody KPL-1 but not by TB5, since the epitope recognized by this antibody is essential for its interaction with P-selectins and L-selectins in humans, completely blocking the recognition of PSGL-1 by selectins [46].

These analyses provided evidence that PSGL-1 is functionally active in ALCL clones. Interfering with cell adhesion, it is possible to maintain cancer cells in circulation and enhance drug cytotoxic effect; moreover, anti-PSGL1 antibodies can induce cell death, causing apoptosis after direct stimulation of ALCL cells through the antigen or activating the immune system. In this study, we focused our test on ADCC and CDC. As a proof of concept of the potential use of anti-PSGL antibodies, mouse mAbs were tested, but murine Fc only partially interacted with receptors (Fc receptors, C1q, etc.) of human effector systems. The evidence that TB5, more than KPL-1, was able to kill ALCL cell lines prompted us to develop fully human or chimeric recombinant antibodies against PSGL1 in order to fully exploit the induction of CDC but also ADCC and phagocytosis. The ideal anti-PSGL1 antibody should have the capacity to neutralize PSGL1 interaction with selectins as described for KPL1 but also to guarantee cell cytotoxicity by apoptosis or caused by the immune system as shown for TB5.

Finally, data suggested that anti-PSGL-1 antibodies are potential candidates for the development of an Ab-based approach in ALCL therapy, alone or in combination with anti-CD30 approaches.

## 5. Conclusions

In conclusion, our data identify *SELPLG* and its coded protein, PSGL-1, as a marker of ALCL. PSGL-1 may play a central role in CD30+ ALCL, where its expression correlates with that of CD30 itself. It might replace TCR signaling in this TCR deficient setting, suggesting that Ab-based immunotherapy with anti- PSGL-1 antibodies is worth to be explored for ALCL.

## Figures and Tables

**Table 1:** Information about the antibodies used in IHC.

Antigen	Host	Clonality	Clone	Code Number	Source	Dilution	pH
CD162 (PSGL-1)	Mouse	Monoclonal	KPL-1	SC-13535	Santa Cruz, Dallas, United States	1:100	6
CD62P (P-Selectin)	Mouse	Monoclonal	C34	CD62P-367	Leica Biosystems, Newcastle Ltd., Newcastle, UK	1:50	9
CD62E (E-Selectin)	Mouse	Monoclonal	16G4	CD62E-382	Leica Biosystems, Newcastle Ltd., Newcastle, UK	1:25	9
CD62L (L-Selectin)	Rabbit	Polyclonal	n.a.	ab135792	Abcam, Cambridge, UK	1:50	9
SYK	Rabbit	Polyclonal	n.a.	ab155187	Abcam, Cambridge, UK	1:100	9

Abbreviations: n.a. = not available.

**Table 2:** Samples, comprising malignant and non-malignant T- and B-cells, used in GEP analysis.

T-Cell Setting (*n* = 180)	B-Cell Setting (*n* = 498)
AITLs (40)	MMs (212)
PTCLs, NOS (67)	MCLs (78)
ALCLs, ALK+ (26)	FLs (45)
ALCLs, ALK- (10)	BLs (41)
ATLLs (13)	DLBCLs (31)
Reactive tissue (4)	SMZLs (14)
Normal T-cell samples (20)	CHLs (12)
	CLLs (10)
	NLPHLs (5)
	PMBCLs (5)
	THRLB-CLs (4)
	Normal B-cell samples (41)

Abbreviations: AITLs, angioimmunoblastic T-cell lymphomas; PTCLs, NOS, peripheral T-cell lymphomas, not otherwise specified; ALCLs, anaplastic large-cell lymphomas; ALK, anaplastic lymphoma kinase; ATLLs, adult T-cell leukemia/lymphomas; MMs, multiple myelomas; MCLs, mantle cell lymphomas; FLs, follicular lymphomas; BLs, Burkitt lymphomas; DLBCLs, diffuse large B-cell lymphomas; SMZLs, splenic marginal zone lymphomas; CHLs, classical Hodgkin lymphomas; CLLs, B-cell chronic lymphocytic leukemias; NLPHLs, nodular lymphocyte-predominant Hodgkin lymphomas; PMBCLs, primary mediastinal large B-cell lymphomas; THRLB-CLs, T-cell/histiocyte-rich large B-cell lymphomas.

**Table 3:** Information about the antibodies used in cellular and functional assays.

Antigen	Host	Clonality	Clone	Code Number	Source
CD162 (PSGL-1)	Mouse	Monoclonal	KPL-1	328802	BioLegend, San Diego, United States
CD162 (PSGL-1)	Mouse	Monoclonal	TB5	21271621	Immunotools, Friesoythe, Germany
CD46	Mouse	Monoclonal	GB24	n.a.	Santa Cruz, Dallas, United States
CD55	Mouse	Monoclonal	BRIC216	n.a.	IGBRL Research Products, Bristol, UK
CD59	Mouse	Monoclonal	YTH53.1	n.a.	Provided by prof. S. Meri (Helsinki, Finland)

Abbreviations: n.a. = not available.

## Data Availability

The data presented in this study are available within the article and in the Supplementary Materials.

## Supplementary Materials

The following are available online at https://www.mdpi.com/article/10.3390/cancers13122958/s1, Figure S1: SELPLG gene expression is higher in ALCL samples, Figure S2: SELPLG gene expression in B-cell setting, Figure S3: PSGL-1 expression and distribution in ALCLs and PTCLs, NOS, Figure S4: Gene correlation analysis with *SELPLG* expression in B-NHL, in CHL, non-neoplastic samples and ALCL settings, Figure S5: The significant correlation between *SELPLG* and *TNFRSF8* gene expression was confirmed in B-NHLs, Figure S6: Network analysis by GeneMANIA, Figure S7: A detailed analysis of *SELPLG* and TCR effector genes correlation, Figure S8: P-, E- and L-selectins expression in prototypical cases of ALCL, Figure S9: FACS analysis on ALCL cell lines, Table S1: IHC SELPLG expression in ALCLs, PTCLs, NOS and ALCL/PTCL, NOS using a semiquantitative approach, Data S1: A detailed analysis encompassing all genes of the reported correlations, Data S2: The GO analysis about the genes related to *SELPLG* gene, Data S3: A detailed analysis of all the reported correlations by GeneMANIA.
