# Supplementary material for: Constitutive PSGL-1 Correlates with CD30 and TCR Pathways and Represents a Potential Target for Immunotherapy in Anaplastic Large T-Cell Lymphoma
Source: Cancers (Basel). 2021 Jun 12;13(12):2958. doi: 10.3390/cancers13122958 (PMC8231564; doi:10.3390/cancers13122958)
Supplement: Supplementary file 1 [file cancers-13-02958-s001.zip › cancers-Supplementary Information.pdf]

cancers

MDPI

Supplementary Materials

Constitutive PSGL-1 Correlates with CD30 and TCR Pathways and Represents a Potential Target for Immunotherapy in Anaplastic Large T-Cell Lymphoma

Beatrice Belmonte, Valeria Cancila, Alessandro Gulino, Mohsen Navari, Walter Arancio, Paolo Macor, Andrea Balduit, Sara Capolla, Gaia Morello, Davide Vacca, Ines Ferrara, Giorgio Bertolazzi, Carmela Rita Balistreri, Paolo Amico, Federica Ferrante, Antonino Maiorana, Tiziana Salviato, Pier Paolo Piccaluga and Alessandro Mangogna

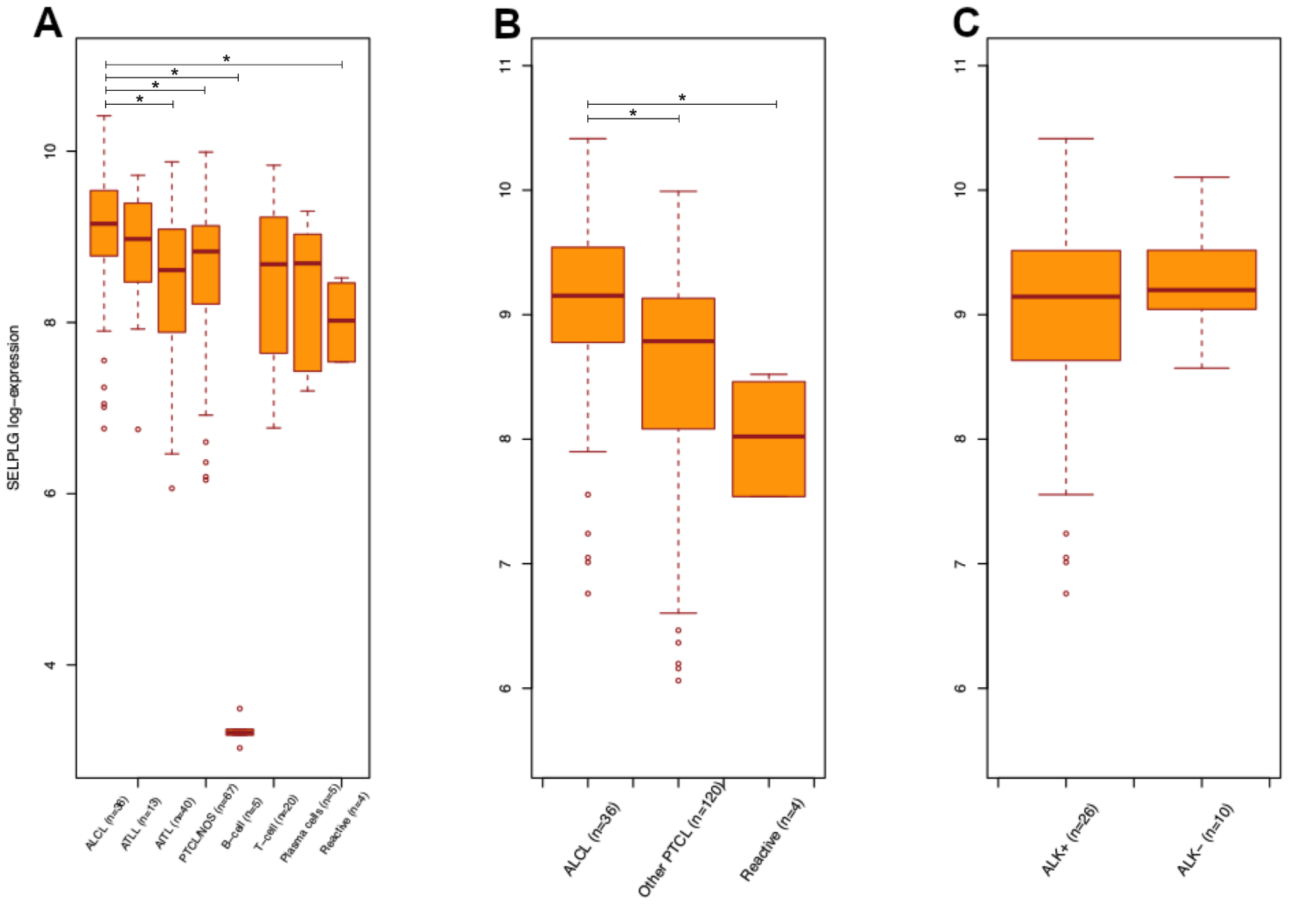

Figure S1. *SELPLG* gene expression is higher in ALCL samples. (**A**) *SELPLG* gene expression in ALCLs was compared to several other cell types. * = adjusted *p*-value < 0.05. (**B**) Higher *SELPLG* gene expression in ALCLs was compared to other PTCL lymphomas and reactive tissues. * = adjusted *p*-value < 0.05. (**C**) No significant differences were detected in *SELPLG* gene expression between ALK+ and ALK- ALCLs.

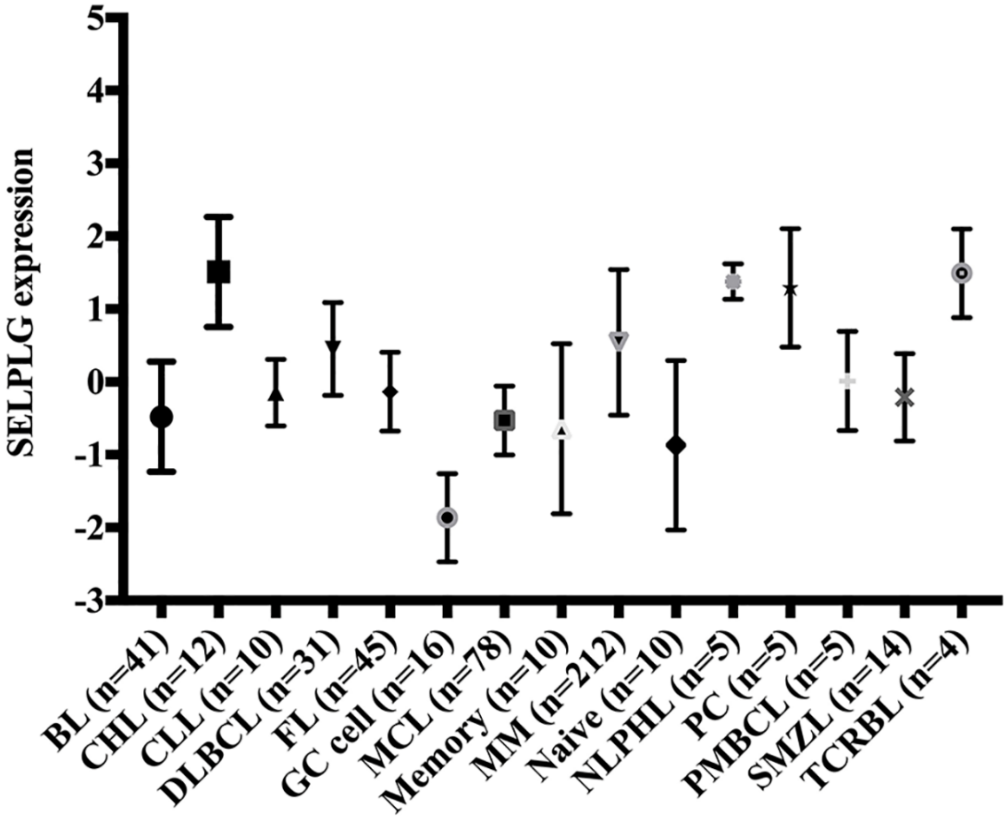

Figure S2. *SELPLG* gene expression in B-cell setting**.** Significantly higher *SELPLG* gene expression was detected in plasma cells and related neoplasms.

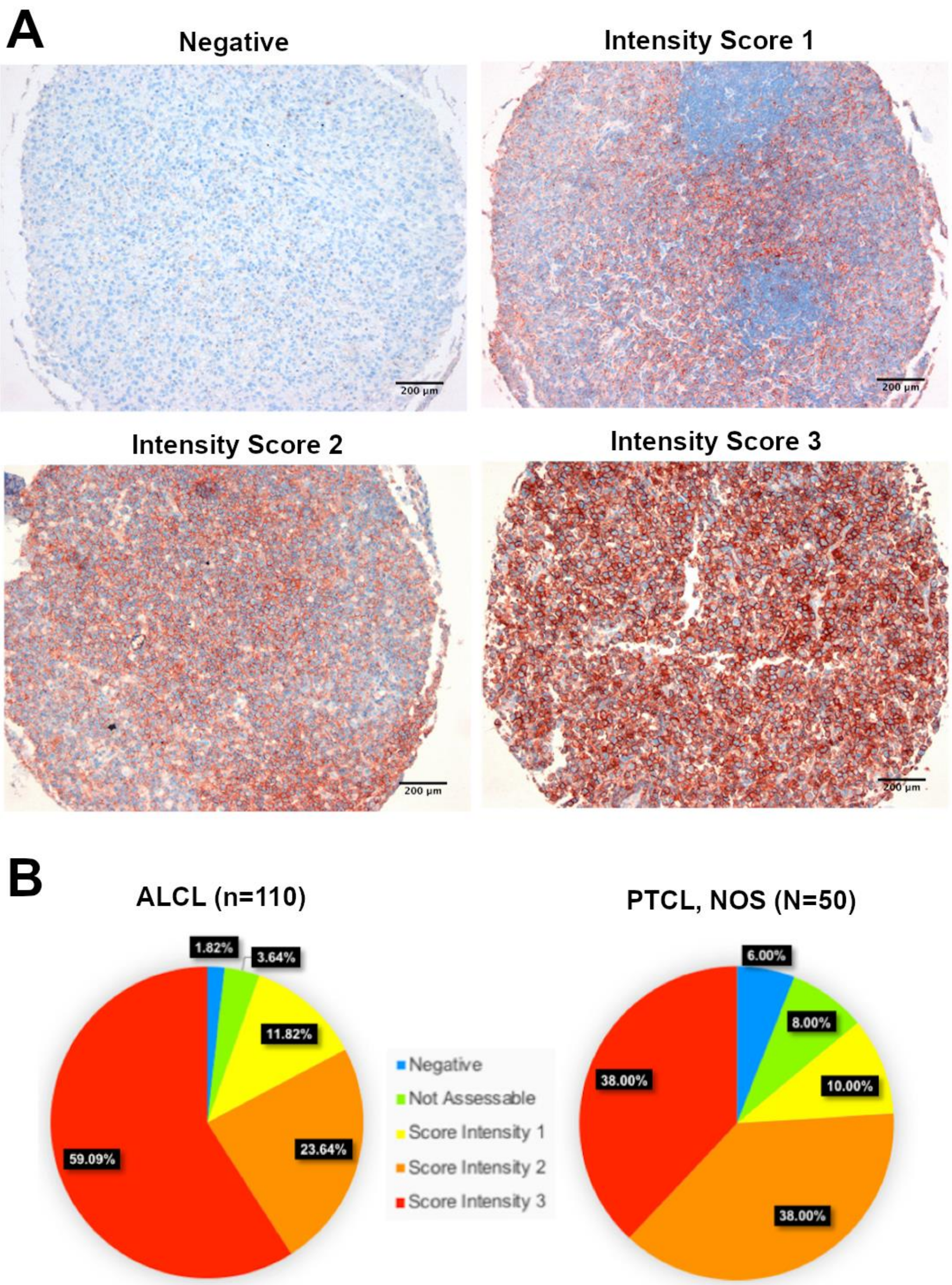

Figure S3. PSGL-1 expression and distribution in ALCLs and PTCLs, NOS. (**A**) Representative microphotographs of IHC score intensity of PSGL-1 expression. Scale bar 200 µm. (**B**) Pie charts of PSGL-1 protein expression in ALCLs and PTCLs, NOS using a semiquantitative immunohistochemical approach.

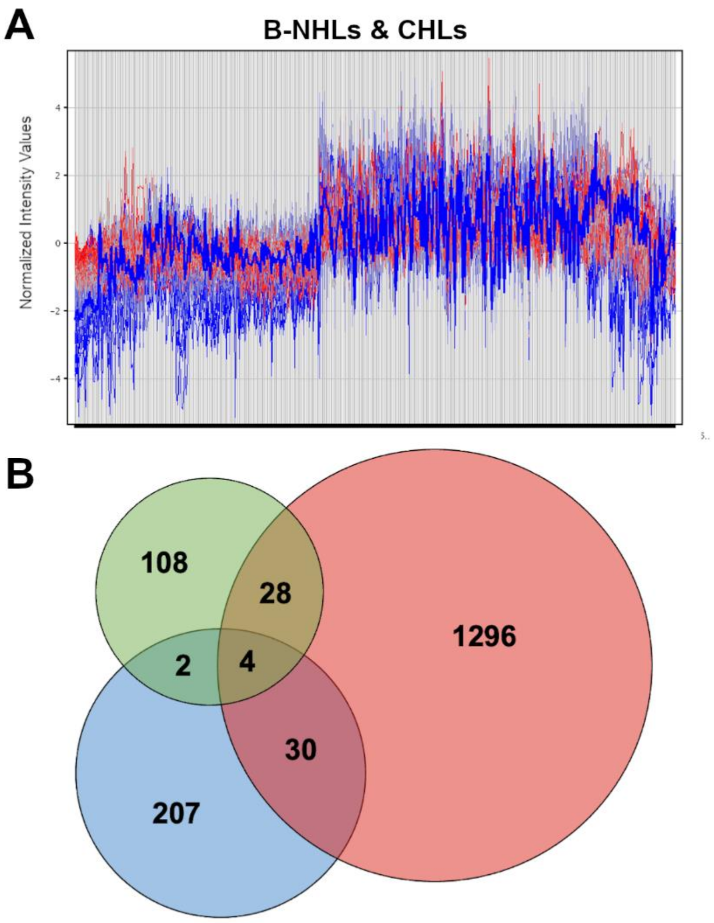

Figure S4. Gene correlation analysis with *SELPLG* expression in B-NHL, in CHL, non-neoplastic samples and ALCL settings. (**A**) GEP analysis showed genes with a positive Pearson product-moment correlation coefficient (0.50 to 1) with *SELPLG* gene expression in B-NHLs and CHLs. (**B**) Genes with a positive correlation with *SELPLG* expression in ALCLs (red), B-NHLs and CHLs (cyan) and non-neoplastic samples (green). In B-settings, 142 genes correlated with *SELPLG* gene expression; only 32 genes overlapped with those emerging from the ALCL analysis and 6 with non-neoplastic samples.

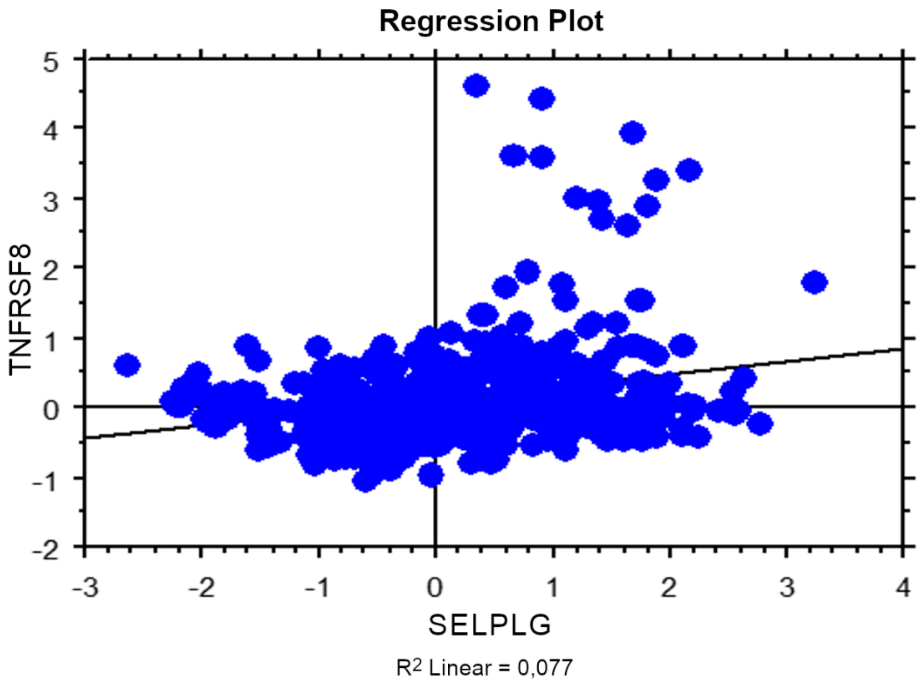

Figure 5. The significant correlation between *SELPLG* and *TNFRSF8* gene expression was confirmed in B-NHLs. The positive correlation between the expression of the two genes have been report by the analysis on the B-cell setting. *p*-value < 0.01..

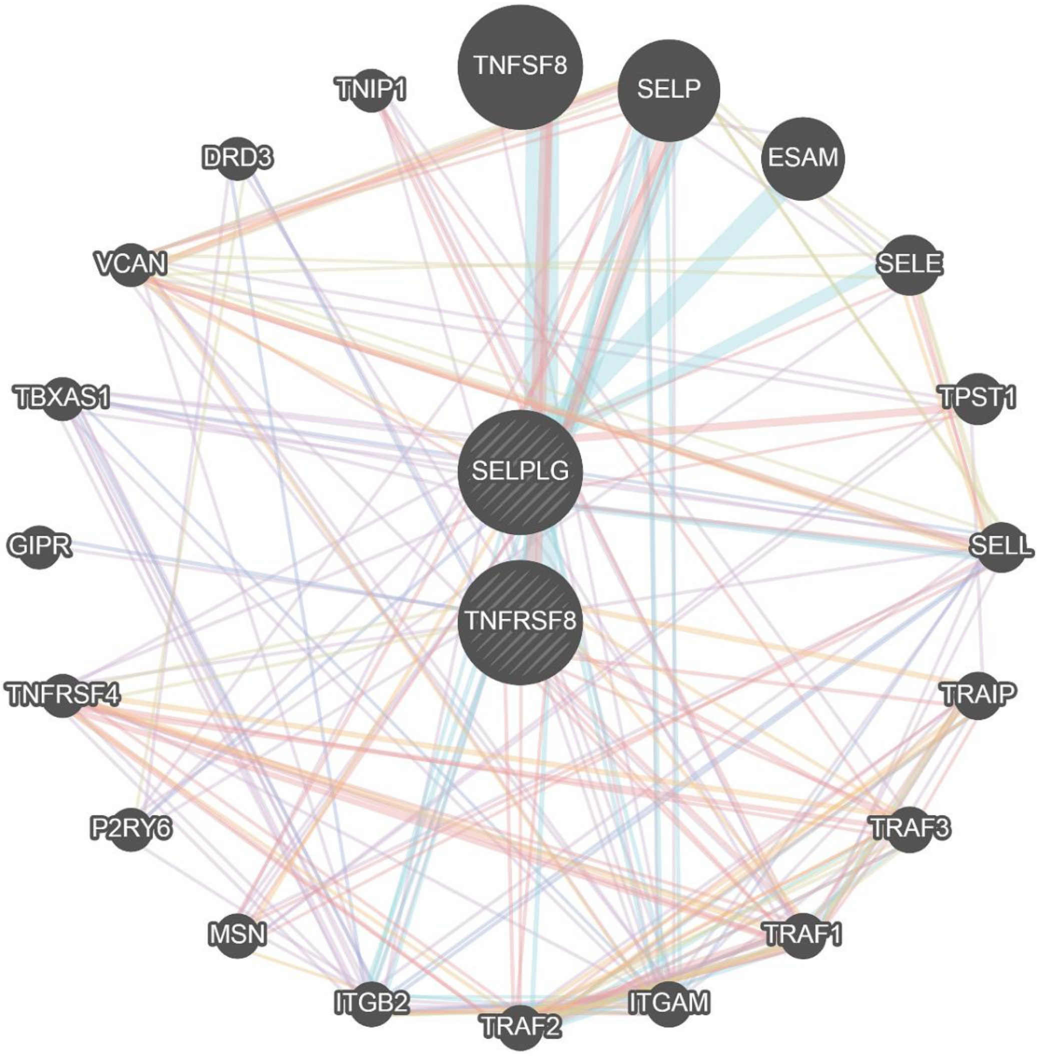

Figure 6. Network analysis by GeneMANIA. Six genes potentially correlated with both *SELPLG* and *TNFRSF8*.

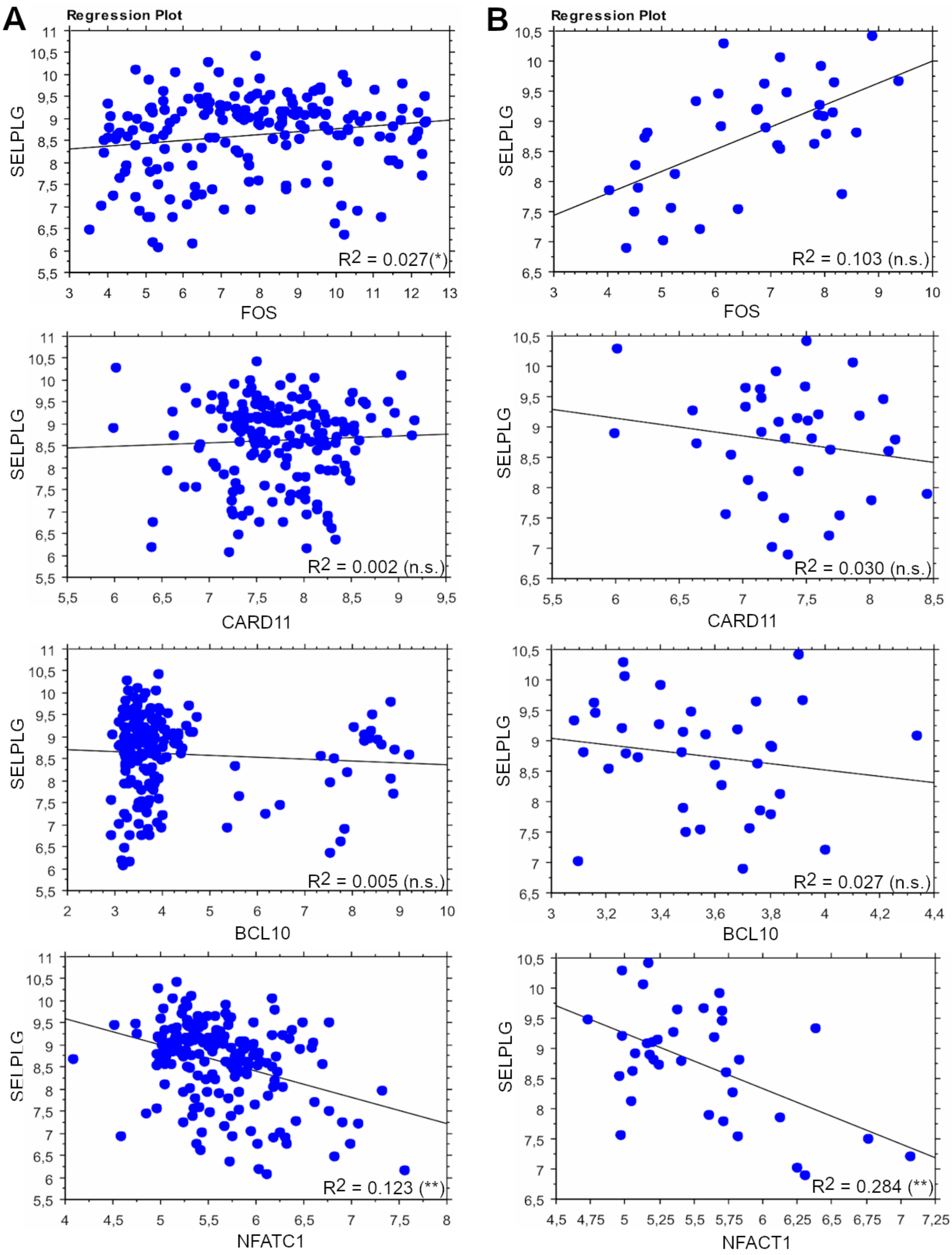

Figure S7. A detailed analysis of *SELPLG* and TCR effector genes correlation. Expression of *FOS*, *JUN*, *NFATC1*, *CARD11* and *BCL10* genes in correlation with that of *SELPLG* in the whole T-cell setting (**A**), and in ALCLs only (**B**). *p*-values: n.s. = not significant; * = < 0.05; ** = < 0.01.

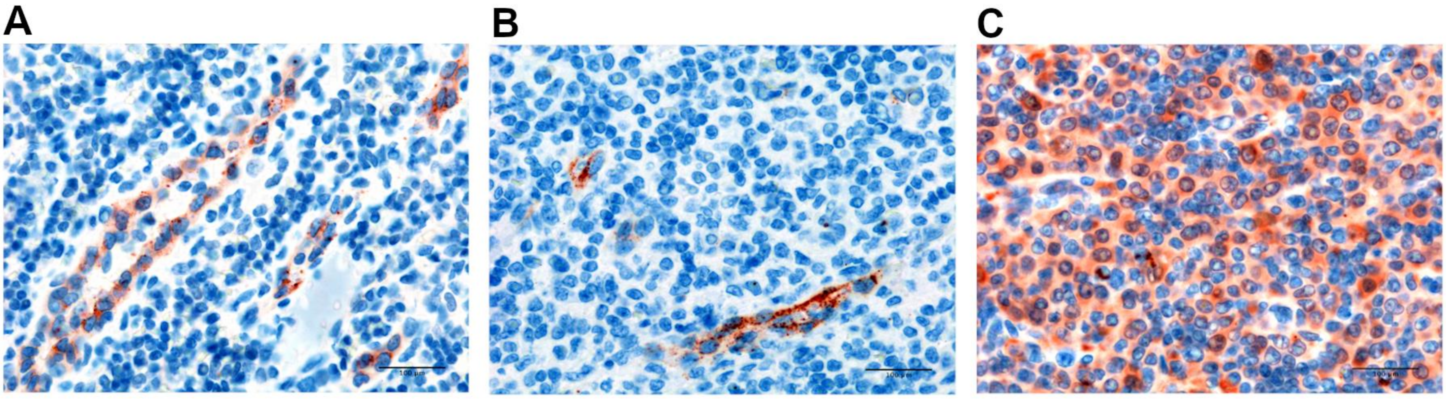

Figure S8. P-, E-, and L-selectins expression in prototypical cases of ALCL. (**A**) P-selectin in an ALCL case that is relatively low for SELPLG staining. **(B)** E-selectin in an ALCL case that is relatively low for SELPLG staining. **(C)** L-selectin in an ALCL case that is relatively low for SELPLG staining. Scale bar 100 µm.

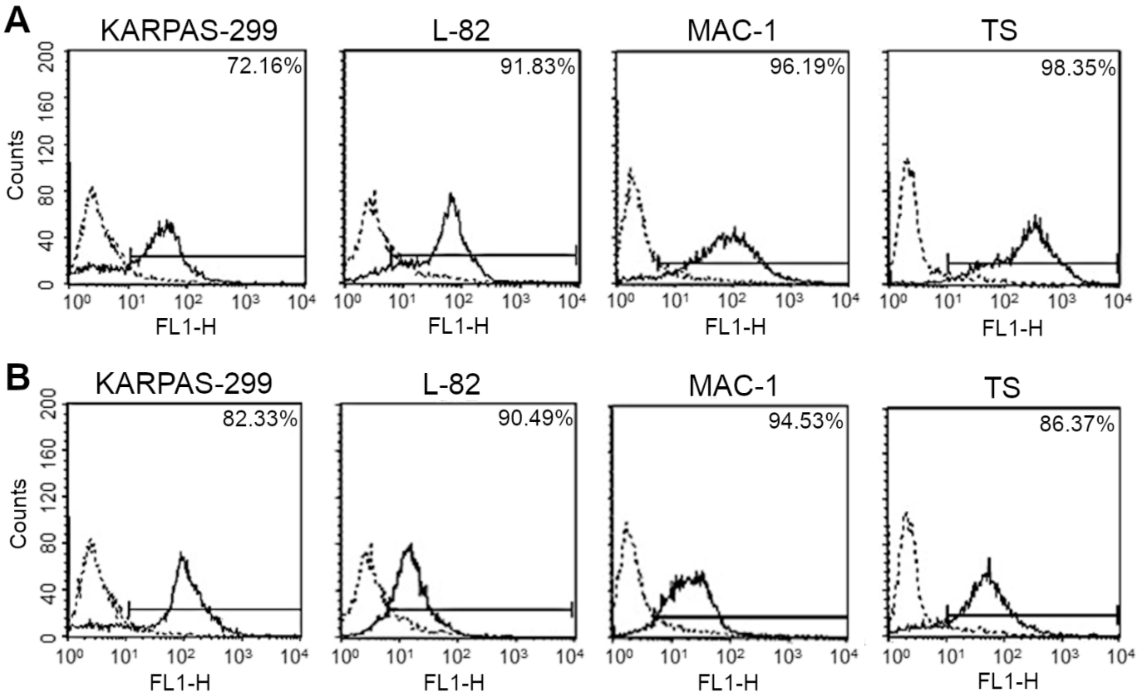

Figure S9. FACS analysis on ALCL cell lines**.** Four ALCL cell lines (KARPAS-299, L-82, MAC-1 and TS) were analyzed by flow cytometry using two independent anti-PSGL1 antibodies, KPL-1 (**A**) and TB5 (**B**).

Table S1. IHC SELPLG expression in ALCLs, PTCLs, NOS and ALCL/PTCL, NOS using a semiquantitative approach.

	Positive	Negative	Not Assessable	Score Intensity 1	Score Intensity 2	Score Intensity 3	Median Intensity
ALCLs *n* = 110	104 (94%)	2 (1.8%)	4 (3.6%)	13 (11.8%)	26 (23.6%)	65 (59%)	3
PTCLs, NOS *n* = 50	43 (86%)	3 (6%)	4% (8%)	5 (10%)	19 (38%)	19 (38%)	2
ALCLs/PTCLs, NOS *n* = 160	92%	3.1%	5%	11%	28%	52.5%	3

Data S1. A detailed analysis encompassing all genes of the reported correlations.

Data S2. The GO analysis about the genes related to *SELPLG* gene.

Data S3. A detailed analysis of all the reported correlations by GeneMANIA.
